# Presenteeism among self-employed workers: Korean working conditions survey

**DOI:** 10.1186/s40557-014-0032-1

**Published:** 2014-10-01

**Authors:** Min-Su Kim, Jae Bum Park, Kyoung-Bok Min, Kyung-Jong Lee, Kimin Kwon

**Affiliations:** 1 Department of Occupational and Environmental Medicine, Ajou University Hospital, Suwon, South Korea

**Keywords:** Self-employed, Presenteeism, Korean working conditions survey, Absenteeism

## Abstract

**Objective:**

Presenteeism has become a public concern recently. Thus, we aimed to understand the relationship between self-employed workers and presenteeism using a nationally representative sample of Korean workers.

**Methods:**

Using data from the Korean Working Conditions Survey conducted in 2011, a total of 43,392 workers including paid employees and self-employed workers were analyzed. The effect of employment status on presenteeism was analyzed using logistic regression analysis. The independent variables were socioeconomic characteristics, working conditions, and working environments.

**Results:**

Among the 43,392 workers, 34,783 were paid and 8,609 were self-employed. Self-employed workers were more likely to exhibit presenteeism than were paid workers. An elevated odds ratio of 1.27 (95% CI 1.19-1.36) was found for presenteeism among self-employed workers.

**Conclusion:**

Being self-employed was significantly related with exhibiting presenteeism. Additional research should investigate whether other factors mediate the relationship between employment status and presenteeism as well as ways to reduce presenteeism among self-employed workers.

## Introduction

The definition of ‘presenteeism’ varies in the literature. Traditionally, its definition has been focused on the economic impact made by someone who worked while being ill [1]. Accordingly, the Stanford Presenteeism Scale has been used to evaluate how health problems may affect performance or productivity [2]. Recently, however, a behavioral definition that describes the action of working while being ill has been employed to describe presenteeism. Simpson [3] defined presenteeism as a tendency to continue working over the effective time for work and Aronsson [4] defined it as a worker who feels unhealthy yet still participates in work activities. Moreover, others have defined it as when someone works longer and rests shorter hours than their workplace recommends for fear of losing employment [5]. Sanderson [6] defined presenteeism in terms of its economic and behavioral impact, but clinicians tend to place more weight on the behavioral aspect than the economic one. In addition, presenteeism was found to be correlated with stress or the potential for absence, but not with reductions in efficiency and output, and is likely to be regarded as a risk factor for workers’ health problems [7].

Moon-Hee Joung et al. [8] studied a correlation between presenteeism and the stress suffered by of workers at small and medium-sized companies and reported that both work stress and sociopsychological stress affected workers’ health and was related to decreased job performance. In addition, Mi-Sook Gun et al. (2011) reported a positive correlation between presenteeism and the stress suffered by nurse practitioners, thus work stress was inversely related to job performance [9]. Kivimaki et al. (2005) studied the behavioral aspect of presenteeism by comparing workers’ attendance with their risk for cardiovascular diseases according to the prevalence of severe coronary artery diseases and found that the prevalence to be two times higher among workers who never missed a day of work than that among those who were sometimes absent [10].

Studies on presenteeism have been conducted mostly on nurses, workers at small and medium-sized companies, and paid workers. However, South Korea’s recent economic conditions indicate that not only paid workers but also self-employed workers are in danger of developing the behavioral definition of presenteeism. According to Statistics Korea, the percentage of self-employed people had decreased for 20 years in the economically active population, but has rebounded since the second half of 2011, particularly among those older than 50 [11]. Moreover, it has been predicted that adults older than 50 and living in Seoul will comprise a higher percentage of self-employed workers than they previously did, and the mandatory retirement of baby boomers (born between 1955 and 1963) may further increase the number of adults who become self-employed [12]. Increases in the number of self-employed people may lead to an increase in poor working conditions that are likely to cause health problems. Self-employed workers are legally considered the employer, therefore have no basic health insurance coverage, except for employment insurance or industrial accident compensation insurance [13],[14]. Moreover, these workers must accept responsibility for all possible risks in the workplace, unlike paid workers [13],[14]. Taking a day off directly influences the income of a self-employed worker; thus, they may be more likely to exhibit presenteeism than are paid workers. Nevertheless, previous studies have tended to investigate this relationship among paid workers, and very few studies have considered the effect of the presenteeism on self-employed people. One reason for this may be that the concept of presenteeism was created from the economic perspective of paid workers’ productivity. Moreover, the health problems of self-employed workers in South Korea have not been well studied. Therefore, we aimed to understand the relationship between employment, paid workers vs. self-employed workers, and presenteeism and the affect of sociodemographic variables, working conditions, and working environments on this relationship.

## Materials and methods

### Subjects

This study used data from the 2011 Korean Working Conditions Survey that is the third survey of the Occupational Safety and Health Research Institute), following those from 2006 and 2010. Data were conducted on employees older than 15 years living in 16 cities or provinces (including Jeju Island) in South Korea, and trained interviewers collected data from each respondent. In total, the 50,032 respondents were classified as either employers, self-employed workers, paid workers, unpaid family workers, or others (workers in a special occupational category, for example, bus driver, home school teacher). A self-employed worker was defined as someone who operates their own business alone, with the help of unpaid family workers, or without the aid of regular employees, and an employer (business owner) was defined as someone who employs at least one regular employee. A paid worker refers to someone who is contracted with another paid worker, individual, household, or company and is compensated for his or her work.

In the literature, no consistent definition of employers or self-employed workers exists. The Organization for Economic Cooperation and Development regards employers, own account workers, members of producer cooperatives, and unpaid family workers as self-employed workers, in conformity with the definition of International Labour Organization (ILO) [15]. Statistics Korea defines employers, own account workers, and unpaid family workers as self-employed workers, and names both employers and own account workers as ‘self-employed owners’ in general [16]. However, these definitions do not fully explain the general characteristics of self-employed workers. For example, sometimes employers and own account workers fall under the same category, but in the strict sense, employers can be distinguished from own account workers in terms of their income and education level. Self-employed workers, which have increased in South Korea, refer to people whose work are the only means to their livelihood, most of whom have no other employees [17]. Thus, self-employed workers in this study refer to people who run their own businesses without any other employees are distinguished from paid workers. We included data from 43,392 respondents, excluding 6,509 who were employers, unpaid family workers, or others and 131 who refused provide their average monthly income.

### Methods

Respondents were asked whether they had ever worked while being ill in the past 12 months. Those who answered yes were regarded as being exposed to presenteeism.

Gender, age, education level, average monthly income, smoking, and drinking alcohol were used as covariates. Smoking was classified as ever or never smokers. In addition, respondents were classified as either heavy drinkers (more than 3 cups of soju, Korean distilled liquor), moderate drinkers (less than 3 cups of SOJU), and nondrinkers according to the guidelines by the World Health Organization [18].

Working conditions were analyzed based on the number of hours worked per week, their level of satisfaction with their working environments, and their subjective health status as variables that might influence presenteeism. The number of hours worked per week were classified into four categories according to the Labor Standards Act [19], as working less than 40 hours a week, 40 to 48 hours a week, 49 to 60 hours a week, or more than 60 hours a week.

Work hazards were categorized as physical, chemical, biological, ergonomic, and emotional hazards. Respondents were asked how often they are exposed to physical, chemical, biological, and ergonomic factors while working. Exposures to ergonomic factors were collected from one of seven choices as follows: (1) throughout the entire work, (2) throughout nearly all of the work, (3) throughout three-fourths of the work, (4) throughout half of the work, (5) throughout one-fourth of the work, (6) hardly exposed, or (7) never exposed. The respondents who answered either answers one through five, as listed above, were regarded as being exposed to work hazards. Exposure to emotional factors were collected as either always, very often, sometimes, hardly, or never, and respondents who answered always, very often, or sometimes were regarded as being exposed to emotional hazards. Physical hazards were exposures to vibrations from hand tools or machinery; noises that cause people to communicate in a very loud voice; working in a high temperature that induces sweating even during a break; low indoor and outdoor temperatures; or the inhalation of smoke, fume, powder or dust. Chemical hazards included the inhalation of vapor from organic solvents such as thinners, handling or being exposed to chemical products or materials, or being exposed to secondhand smoke. Biological hazards included the handling of or direct contact with infectious materials such as wastes, body fluids, and/or experimental materials. Ergonomic hazards were being exposed to a tiring or painful posture, having to lift or move people, having to lift or move heavy things, standing or walking for extended periods, or performing repetitive hand or arm movements. Emotional hazards included dealing with customers, passengers, students, or patients, namely people beside coworkers; dealing with angry customers or patients; being aware of their work requiring emotional endurance; or needing to hide their emotions at work. The previously mentioned definitions were adapted from a previous study on working environments [20].

### Data analysis

Paid workers were analyzed separately form self-employed workers according to the respondents’ self-reported employment status, and differences between these workers with respect to sociodemographic characteristics, working conditions, and working environments were investigated. After an adjustment for sociodemographic characteristics, working conditions, and working environments, a logistic regression analysis was conducted to determine if any sociodemographic characteristics, working conditions, or working environments were associated with presenteeism. All analyses were performed using SPSS for Windows version 19.0 (SPSS Inc., Chicago, IL, USA).

## Results

### The sociodemographic characteristics and working conditions of paid and self-employed workers

Out of the 43,392 total respondents, 34,783 were paid workers and 8,609 were self-employed workers. There were more men who were self-employed than men who were paid workers (66.6% vs. 59%) and the same trend was found for smoking. In addition, the percentages of moderate drinkers and highly educated people were higher among self-employed workers than that among paid workers. However, the average age was lower among the paid workers than self-employed workers were. Additionally, paid workers tended to be more satisfied with their working environments (73.1% vs. 67.4%) and more likely to work a 40 to 48-hour workweek than self-employed workers were. The majority of self-employed workers worked 48 hours or more a week. Moreover, paid workers tended to report a better subjective health status than self-employed workers did (97.1% vs. 91.5%). For presenteeism, 20.6% and 28.9% of paid workers and self-employed workers were regarded as being exposed to presenteeism, respectively (Tables 1 and 2).

**Table 1:** **Characteristics of the study population by employment status** ***n (%)***

	**Paid workers**	**Self-employed workers**	P-value*
Total			
Age			
15-29	7387 (21.2)	216 (2.5)	<0.001
30-39	9879 (28.4)	887 (10.3)	
40-49	9260 (26.6)	2197 (25.5)	
50-59	5902 (17.0)	2704 (31.4)	
≥60	2355 (6.8)	2605 (30.3)	
Sex			
Male	20531 (59.0)	5737 (66.6)	<0.001
Female	14252 (41.0)	2872 (33.4)	
Income (10,000 won/month)			
<100	4263 (12.3)	1566 (18.2)	<0.001
100-199	13703 (39.4)	3008 (34.9)	
200-299	9728 (28.0)	2389 (27.8)	
≥300	7090 (20.4)	1646 (19.1)	
Education			
<Elementary	280 (8.0)	426 (4.9)	<0.001
Elementary school	1127 (3.2)	1035 (12.0)	
Middle school	2279 (6.6)	1431 (16.6)	
High school	12762 (36.7)	3867 (44.9)	
College	6390 (18.4)	820 (9.5)	
University	10960 (31.5)	958 (11.1)	
Graduate school	984 (2.8)	73 (0.8)	
Alcohol intake			
Heavy	7891 (22.7)	2642 (30.7)	<0.001
Moderate	5226 (15.0)	1353 (15.7)	
None	21666 (62.3)	4615 (53.6)	
Smoking			
Never or past	22864 (65.7)	5733 (66.6)	0.133
Current	11918 (34.3)	2876 (33.4)	

*Calculated using the chi-square test.

**Table 2:** **Comparison of the working conditions and environments between paid and self-employed workers** ***n (%)***

	**Paid workers**	**Self-employed workers**	P-value*
Presenteeism			<0.001
Yes	7180 (20.6)	2485 (28.9)	
No	27603 (79.4)	6124 (71.1)	
Subjective health status			
Good	33772 (97.1)	7879 (91.5)	<0.001
Bad	1011 (2.9)	731 (8.5)	
Working condition			
Average hours worked per week			
<40	3428 (9.9)	1263 (14.7)	<0.001
40-48	18507 (53.2)	1525 (17.7)	
49-60	9734 (28.0)	3101 (36.0)	
≥61	3114 (9.0)	2720 (31.6)	
Satisfaction of working conditions			
Good	25422 (73.1)	5805 (67.4)	<0.001
Bad	9361 (26.9)	2804 (32.6)	
Working environments			
Physical factors			
Noise requiring workers to speak in loud voice			
Yes	7793 (22.4)	1831 (21.3)	0.023
High temperatures causing perspiration even not working			
Yes	6262 (18.0)	2506 (29.1)	<0.001
Chemical factors			
Inhaling vapors such as solvents and thinners			
Yes	2196 (6.3)	444 (5.2)	<0.001
Handling or being in skin contact with chemical products or substances			
Yes	2551 (7.3)	970 (11.3)	<0.001
Ergonomic factors			
Working in tiring or painful positions			
Yes	17427 (50.1)	5503 (63.9)	<0.001
Lifting or moving people			
Yes	3269 (9.4)	746 (8.7)	0.036
Carrying or moving heavy loads			
Yes	12041 (34.6)	4736 (55.0)	<0.001
Standing or walking for extended periods of time			
Yes	21077 (60.6)	6052 (70.3)	<0.001
Repetitive hand or arm movements			
Yes	23516 (67.6)	6397 (74.3)	<0.001
Emotional factors			
Working with angry customers, passengers, pupils, patients, etc.			
Yes	5583 (16.1)	1296 (15.1)	0.023
Having to hide your feelings at work			
Yes	22008 (63.3)	5197 (60.4)	<0.001

*Calculated using the chi-square test.

### Working conditions of paid and self-employed workers

For physical hazard exposure, self-employed workers were more likely to be exposed to vibrations from hand tools or machinery (30.7% vs. 25.9%), high temperatures that induced sweating even during a break (72.0% vs. 26.3%), low indoor and outdoor temperatures (16.0% vs. 11.8%), and inhalation of smoke, fumes, powder, or dust (22.4% vs. 20.1%) than paid workers were. However, paid workers were more likely to be exposed to noises that caused people to communicate in a very loud voice (22.4% vs. 20.7%). For chemical hazards, self-employed workers were more likely to handle or be exposed to chemical products or materials (10.0% vs. 7.3%) and secondhand smoke (11.8% vs. 10.7%) than paid workers were. Paid workers were more likely to inhale vapor from organic solvents such as thinners (6.3% vs. 4.9%) and handle or be exposed to infectious materials such as wastes, body fluids, and experimental (4.0% vs. 2.1%) than self-employed workers were. However, self-employed workers tended to report a higher exposure to all of the surveyed items except having to lift or move people (9.4% vs. 8.0%) than paid workers were. Moreover, self-employed workers tended to experience more emotional hazards such as having to deal with customers, passengers, students, or patients, namely people beside coworkers (60.8% vs. 51.8%)’ and exhibit emotional endurance at work (42.9% vs. 41.2%) than paid workers were. Nonetheless, paid workers tended to deal with angry customers or patients (16.1% vs. 15.9%) and hide their emotions at work (63.3% vs. 62.5%) more than self-employed workers did (Table 2). Although differences in the working conditions and environments among self-employed workers and paid workers were found, our logistic regression analysis did not reveal any significant odds ratios (OR) for the following exposure variables: vibrations from hand tools or machinery, low indoor and outdoor temperatures, inhalation of smoke, fumes, powder, or dust, secondhand smoke, handling of or exposure to infectious materials such as wastes, body fluids, and experimental materials, ‘dealing with customers, passengers, students or patients, namely people beside coworkers, and having to exhibit emotional endurance at work (data not shown).

### Relationship between employment status and presenteeism

To understand the relationship between employment status and presenteeism, ORs and the 95% confidence intervals (CI) were calculated using logistic regression analysis. We found that self-employed workers had a significantly higher OR before and after adjustment for sociodemographic characteristics and working conditions (crude OR: 1.56, 95% CI: 1.48 – 1.65; adjusted OR 1.27, 95% CI 1.19 – 1.36) (Table 3).

**Table 3:** Odds ratios (OR) of presenteeism by employment type

	**Presenteeism**			
	**Crude OR**	**95% CI**	Adjusted OR*	**95% CI**
Employment type				
Paid worker	1.00	(Reference)	1.00	(Reference)
Self-employed worker	1.56	1.48-1.65	1.27	1.19-1.36

*Adjusted for gender, age, education, income, alcohol intake, smoking, hours worked per week, subjective health status, and all variables estimating one’s working condition and physical, chemical, biological, ergonomic, and emotional factors.

## Discussion

Presenteeism is the opposite of absenteeism and has recently appeared in the literature. Many recent studies have shown that presenteeism is a risk factor for negative health factors and consumes more economic cost than an absence due to sickness does [21]. Considering these economic and physical hazards, we performed this study on presenteeism in paid workers and self-employed workers, especially since many self-employed workers are not properly covered by the social safety net as much as paid workers tend to be [22]. Accordingly, the presenteeism of self-employed workers should be investigated using public health methodologies.

This study aimed to compare the presenteeism of paid workers with that of self-employed workers using samples large enough to be representative of the Korean work force as well as to understand the relationship between presenteeism and sociodemographic factors, working conditions, and working environments. Our results showed that self-employed workers were more likely to be exposed to presenteeism (crude OR: 1.56, 95% CI: 1.48 – 1.65) than paid workers were. Self-employed workers were found to work longer hours, assess their health status as worse, and were less satisfied with their working environments than paid workers were. In addition, self-employed workers tended to be exposed to more hazards that were physical, chemical, ergonomic, and emotional than paid workers were. Variables that measured the working conditions and environment, which were used in the logistic regression analysis, seemed to affect the prevalence of presenteeism, especially among self-employed workers (adjusted OR: 1.27, 95 CI: 1.19 – 1.36). Moreover, significant differences were found for the subjective health status, working environments, and number of hours worked per week between self-employed and paid workers (data not shown). The ORs were 2.67 (95% CI 2.40-2.97) and 1.69 (95% CI 1.60-1.78) for the effect of subjective health status and working environments, respectively. For the number of hours worked per week, the OR increased as the number of working hours increased when compared with those working less than 40 hours a week (working 40 to 48 hours a week: OR 1.41, 95% CI 1.27 – 1.55; working 49 to 60 hours: OR 1.80, 95% CI 1.63 – 1.99; working 61 hours or longer: OR 2.06, 95% CI 1.84 – 2.29).

Even after adjustment for covariates, the odds of exhibiting presenteeism were higher among self-employed workers than that among paid workers. Therefore, employment status may influence this difference. According to our definition of self-employed workers as people who run their own businesses without their employees, namely own account workers, self-employed workers have no one who can substitute for them if they are unable to work. Our results are consistent with those of recent studies that have reported presenteeism was more prevalent among self-employed workers who have no substitutes than in workers at private companies [23].

In South Korea, few studies have investigated the health impacts on self-employed workers, whereas these studies have been being carried out in other countries. The fatality rate was found to be higher among self-employed workers in the agricultural, retail, and distribution industries than it was for paid workers from the same industries [24]. Moreover, self-employed workers and paid workers showed differences in the number of absences and hospital visits [25]. In a study conducted in South Korea, it was reported that self-employed workers were significantly more likely to have abdominal obesity, hypertension, diabetes, coronary artery calcification and stenosis, all of which heighten the risk of cardiovascular diseases, than office workers were [26]. However, some studies reported that self-employed workers paid workers experience similar work-related stress, hence the need for future research on self-employed workers [27].

In our study, paid workers were more satisfied with their working environments than self-employed workers were, and self-employed workers showed more cases of working 49 hours or longer a week than paid employees did. Similarly, paid workers assessed their subjective health status better than self-employed workers did, which is consistent with a previous study that reported presenteeism as being associated with a general health assessment [28]. Therefore, working conditions may negatively affect workers’ health.

The presenteeism of workers can create stress and may be related to cardiovascular diseases, pessimistic results on one’s subjective health assessment, and future absences [10],[29],[30]. Presenteeism is also thought to be associated with mental health [31].

In this study, presenteeism was more prevalent among self-employed workers than that of paid workers, with adjustment for sociodemographic factors and working conditions and environments. Considering the results of previous studies, self-employment might be a risk factor for health problems.

To the best of our knowledge, this is the first study on presenteeism among self-employed Korean. Our findings are noteworthy, but certain limitations should be considered. First, few similar datasets exist for comparison. In addition, this cross-sectional study cannot suggest causality. Moreover, researchers are not in complete agreement on the definition of presenteeism, which impairs the ability to objectively compare presenteeism. Last, no strict definition of what it means to be self-employed exists.

Nevertheless, this study was conducted on large nationwide sample of approximately 50,000 participants thought to be representative of the South Korean work force. Furthermore, highly trained interviewers collected data using a structured questionnaire, thus minimizing information bias. In particular, our respondents were recruited from almost all categories of paid and self-employed workers. We found presenteeism to be significantly more prevalent among self-employed workers than that of paid workers, and this result implies that this may be of importance among self-employed workers, unlike the previous studies that have been focused on the presenteeism of only paid workers. Future studies should further investigate presenteeism and make efforts to solve these problems. Furthermore, structured evaluation tools should be developed along with longitudinal studies to determine the causality and possibly reveal other factors that mediate the relationship between self-employed workers and presenteeism.

## Summary

### Objectives

This study aimed to look into the presenteeism of self-employed workers and determine whether employment status is related to presenteeism. Thus, a comparison was made between the prevalence of presenteeism among self-employed workers and paid workers.

### Methods

Data from the 2011 Korean Working Conditions Survey was used to conduct an analysis on 43,392 workers, which included self-employed workers. Sociodemographic characteristics, working conditions, and working environments were used as covariates, and logistic regression analyses were conducted to identify the correlation between employment status and presenteeism.

### Results

Out of 43,392 respondents, 34,783 were paid workers and 8,609 were self-employed workers. Paid and self-employed workers showed differences in working conditions and environments, and self-employed workers were more likely to exhibit presenteeism than paid workers were (adjusted OR 1.27, 95% CI 1.19 – 1.36).

## Conclusion

This study found that presenteeism was more obvious in self-employed workers than paid workers were even after adjusting for covariates relating to working conditions and environments. Further study is needed to find other factors that may influence the relationship between employment status and presenteeism and to discuss ways to reduce the presenteeism among self-employed workers.

## Competing interests

The authors declare that they have no competing interests.

## Authors’ contributions

M-SK designed the research. KK collected the data. M-SK and JBP performed the statistical analysis. M-SK, K-BM, and K-JL interpreted the data. M-SK wrote the manuscript. All of the authors read and approved the final manuscript.

## References

[B1] Lerner D Amick BC 3rd Rogers WH Malspeis S Bungay K Cynn D The work limitations questionnaire Med Care 2001 39 72 85 10.1097/00005650-200101000-00009 11176545

[B2] Koopman C Pelletier KR Murray JF Sharda CE Berger ML Turpin RS Hackleman P Gibson P Holmes DM Bendel T Stanford presenteeism scale: health status and employee productivity J Occup Environ Med 2002 44 14 20 10.1097/00043764-200201000-00004 11802460

[B3] Simpson R Presenteeism, power and organizational change: long hours as a career barrier and the impact on the working lives of women managers Br J Manag 1998 9 37 50 10.1111/1467-8551.9.s1.5

[B4] Aronsson G Gustafsson K Dallner M Sick but yet at work. An empirical study of sickness presenteeism J Epidemiol Community Health 2000 54 502 509 10.1136/jech.54.7.502 10846192 PMC1731716

[B5] http://www.collinsdictionary.com/dictionary/english/presenteeism?showCookiePolicy=true **Collins English Dictionary.**.

[B6] Sanderson K Cocker F Presenteeism–implications and health risks Aust Fam Physician 2013 42 172 175 23550237

[B7] Leineweber C Westerlund H Hagberg J Svedberg P Alexanderson K Sickness presenteeism is more than an alternative to sickness absence: results from the population-based SLOSH study Int Arch Occup Environ Health 2012 85 905 914 10.1007/s00420-012-0735-y 22270388

[B8] Moon-Hee J Young-Mi L Mikako A Stress and presenteeism in workers of small and medium enterprises Korean J Occup Environ Med 2007 19 47 55 Korean

[B9] Gun MS Choi YH Park KH Job stress and presenteeism of clinical nurses Korean J Occup Health Nurs 2011 20 163 171 Korean 10.5807/kjohn.2011.20.2.163

[B10] Kivimaki M Head J Ferrie JE Hemingway H Shipley MJ Vahtera J Marmot MG Working while ill as a risk factor for serious coronary events: the Whitehall II study Am J Public Health 2005 95 98 102 10.2105/AJPH.2003.035873 15623867 PMC1449859

[B11] Lim J Financial focus: increasing status and countermeasures of self-employed persons over the age of 50 Weekly Financial Brief 2013 22 12 13 Korean

[B12] Forecast of job information Book Forecast of Job Information 2013 The Seoul Metropolitan Government, Seoul

[B13] Lee SR Kim JI Park CI Lee DJ Hong MG Study of self employed labor market Book Study of Self Employed Labor Market 2009 Korea Labor Institute, Korea

[B14] Lee B-H Promoting registration for social insurance? Focusing on insurance premium subsidies Book Promoting Registration for Social Insurance? Focusing on Insurance Premium Subsidies 2011 Korea Labor Institute, Korea

[B15] OECD Factbook 2010 2010 OECD Publishing, Paris

[B16] http://meta.narastat.kr/metasvc/index.do?confmNo=10104 **Economically active population survey.**.

[B17] Kim SB Actual condition and way out of livelihood self-employed Book Actual Condition and way out of Livelihood Self-Employed 2012 Samsung Economic Research Institute, Korea 1 22

[B18] International guide for Monitoring Alcohol Consumption and Related Harm 2000 World Health Organization, Geneva

[B19] http://www.law.go.kr/lsInfoP.do?lsiSeq=152336&efYd=20140324#AJAX **Labor Standards Act.**

[B20] Min KB Park SG Song JS Yi KH Jang TW Min JY Subcontractors and increased risk for work-related diseases and absenteeism Am J Ind Med 2013 56 1296 1306 23794385 10.1002/ajim.22219

[B21] Hemp P Presenteeism: at work--but out of it Harv Bus Rev 2004 82 49 58 155 15559575

[B22] Seo JM Status and Policy Direction of Self-Employed 2011 National Assembly Budget Office, Seoul

[B23] Johansen V Aronsson G Marklund S Positive and negative reasons for sickness presenteeism in Norway and Sweden: a cross-sectional survey BMJ Open 2014 4 e004123 10.1136/bmjopen-2013-004123 24523425 PMC3927796

[B24] Mirabelli MC Loomis D Richardson DB Fatal occupational injuries among self-employed workers in North Carolina Am J Ind Med 2003 44 182 190 10.1002/ajim.10244 12874851 PMC4531843

[B25] Pfeifer C Cyclical absenteeism among private sector, public sector and self-employed workers Health Econ 2013 22 366 370 10.1002/hec.2808 22383260

[B26] Jang KH Park W Kim M Lee D Chae H Moon J Comparison of cardiovascular disease status between large scale industry office and self employed male workers Korean J Occup Environ Med 2011 23 130 138 Korean

[B27] Oren L Job stress and coping: self-employed versus organizationally employed professionals Stress Health 2012 28 163 170 10.1002/smi.1418 22282343

[B28] Taloyan M Aronsson G Leineweber C Magnusson Hanson L Alexanderson K Westerlund H Sickness presenteeism predicts suboptimal self-rated health and sickness absence: a nationally representative study of the Swedish working population PLoS One 2012 7 e44721 10.1371/journal.pone.0044721 22984547 PMC3439368

[B29] Inn-Shil R Dae-Soon J In-Ah K Jae-Hoon R Jong-Uk W Association between job stress, psychosocial well-being and presenteeism, absenteeism: focusing on railroad workers Korean J Occup Environ Med 2012 24 263 273 Korean

[B30] Bergstrom G Bodin L Hagberg J Lindh T Aronsson G Josephson M Does sickness presenteeism have an impact on future general health? Int Arch Occup Environ Health 2009 82 1179 1190 10.1007/s00420-009-0433-6 19504117

[B31] Vahtera J Kivimaki M Pentti J Linna A Virtanen M Virtanen P Ferrie JE Organisational downsizing, sickness absence, and mortality: 10-town prospective cohort study BMJ 2004 328 555 10.1136/bmj.37972.496262.0D 14980982 PMC381046

